# Evaluation of the Developmental Toxicity Induced by E804 in Zebrafish Embryos

**DOI:** 10.3389/fphar.2020.00032

**Published:** 2020-02-14

**Authors:** Rongchun Wang, Kechun Liu, Yun Zhang, Xiqiang Chen, Xue Wang

**Affiliations:** Key Laboratory for Drug Screening Technology of Shandong Academy of Sciences, Key Laboratory for Biosensor of Shandong Province, Biology Institute, Qilu University of Technology, Shandong Academy of Sciences, Jinan, China

**Keywords:** E804, zebrafish, developmental toxicity, oxidative stress, apoptosis

## Abstract

E804, a derivative of indirubin, have multi-biological activities such as anticancer and anti-inflammatory activities, but little is known about its developmental toxicity. In this study, we investigated the toxicity of E804 on the developments of zebrafish embryos. Our results showed that E804 treatment caused a significant increase of the malformation rate compared with the control groups. Pericardial edema and curved body shape were the most morphological abnormalities observed in E804-treated group. The hatching rates and body length of the zebrafish larvae was significantly decreased in E804-treated groups. E804 also affect the development of heart, liver, phagocytes and vascular formation. Further studies showed that the level of reactive oxygen species was significantly increased. The activity of total superoxide dismutase decreased and the concentration of malondialdehyde were increased. Much more apoptotic cells were detected in E804-treated group, compared with the control. In addition, gene-expression results showed that the pathways of oxidative stress and apoptosis were provoked in E804 treated groups. Taken together, our findings will be helpful to understanding E804-induced developmental toxicity and the underlying mechanism.

## Introduction

Indirubin is the an active compound of Chinese herb of indigo blue, which can be used for the treatment of chronic diseases such as chronic myelogenous leukemia, and hepatitis, influenza, and encephalitis (Han, 1994; Wang et al., 2014; Gaboriaud-Kolar et al., 2015). Previous researches have shown that indirubin and its derivatives possessed many biological activities, including anticancer (Ichimaru et al., 2015; Zhang et al., 2015), anti-inflammatory (Mok et al., 2014; Kwok et al., 2016), anti-leukemia, and antivirus effects. The targets of indirubin and its derivatives have been widely studied. Indirubin and its derivatives act as potent inhibitors of CDKs (CDK1 and CDK2) or GSK3 (GSK-3α and GSK3β) (Blazevic et al., 2015; Gaboriaud-Kolar et al., 2015; Saravanan et al., 2019). Indirubin-3′-(2, 3-dihydroxypropyl)-oximether (E804) is an indirubin derivative with potent inhibitory properties targeting CDK2/CycE and STAT3 signaling pathway (Zhang et al., 2015). E804 can modulate immune response with activation of the pro-inflammatory effects in lipopolysaccharide (LPS)-treated macrophages (Babcock et al., 2013). Several studies also showed E804 had the activity of angiogenesis *in vitro* and *in vivo* (Chan et al., 2012; Shin and Kim, 2012). So far, most studies on E804 refer to its activities of anti-tumor, anti-angiogenesis, and so on. However, little is known about the developmental toxicity induced by E804.

Recent studies showed that indirubin derivatives DKP-071 and DKP-073 can activate the apoptotic pathway in cutaneous T-cell lymphoma and melanoma cells by inducing the production of reactive oxygen species (ROS) (Soltan et al., 2019; Zhivkova et al., 2019). Oxidative stress and elevation of ROS induced by chemicals have pleiotropic deleterious effects including developmental toxicity. The defense system of an organism can be destroyed by high level of ROS which can damage the DNA, lipid, and protein macromolecules. Then the dysfunction and the impairment of mitochondria take place and ultimately induce apoptosis (Huang et al., 2018; Jin et al., 2019; Zhu et al., 2019). All of these procedures are toxic to cell growth, division, and differentiation during the development of early embryo (Kumari et al., 2017).

Zebrafish (*Danio rerio*) has been widely accepted as a prominent vertebrate model for toxicological and developmental studies in recent years because of the high fecundity, rapid organogenesis, short generation, and transparent body of embryos and larvae, and so on (Hill et al., 2005; McGrath and Li, 2008; Haque and Ward, 2018; Xia et al., 2018; Al-Samadi et al., 2019; Van Sebille et al., 2019). In addition, it shares high degree of genetic conservation which makes it have additional advantages over traditional animal models (Crawford et al., 2008; Jia et al., 2019). Moreover, one major advantage is that malformation and organogenesis toxicity induced by chemicals can be readily and directly observed under stereo- or fluorescence- microscope at the early developmental stage of zebrafish (Heideman et al., 2005; Sieber et al., 2019).

In this study, zebrafish embryos/larvae were selected as the *in vivo* model to investigate the developmental toxicity of E804. We found that E804 had severe developmental toxicity to zebrafish. The antioxidant capacity, oxidative stress status, and apoptosis levels were assessed. The expression levels of genes related to organogenesis, oxidative stress, and apoptosis were studied after E804 treatment. Our findings will be helpful to understand the developmental toxicity induced by E804 and the underlying molecular mechanisms.

## Material and Methods

### Chemicals and Reagents

E804 was purchased from Enzo Biochem Inc (New York, USA). The compound was dissolved in DMSO to make 5 mM-solution. The treated solutions of E804 were serially diluted in normal bathing medium. All of the other chemicals and regents utilized in the experiments were of analytical grade.

### Zebrafish Husbandry and Embryo Collection

The zebrafish were kept in Zebrafish Drug Screening Platform of Shandong Academy of Science. Zebrafish of wild-type (AB line) and transgenic zebrafish lines [*Tg(cmlc2:EGFP)* (Huang et al., 2003)*, Tg(fli:EGFP)* (Hu et al., 2012)*, Tg(l-fabp:EGFP)* (Her et al., 2003)*, Tg(Lyz : EGFP)* (Hall et al., 2007)] used in this study were feed according to standard procedures. Briefly, fish were kept with constant temperature at 28 ± 0.5°C and 14/10 hour light/dark photoperiod in an aquarium. The fish were fed with live brine shrimp twice a day and dry flakes once a day. The pH value of the water varies from 7.2 to 7.4, and the conductivity varies from 450 to 500 µS/cm. Before mating, adult male and female zebrafish were separated in a breeding tank at the ratio of 2:1. After the light on the next morning, embryos were generated by natural mating. Then the eggs were collected, rinsed three times and cultured in incubator before subsequent experiments. The embryos were checked at 6 hours post fertilization (hpf) under the microscope to make sure all of the embryos stay at the same development stage. At 24 hpf, embryos were examined under the microscope to make sure the lethal rate lower than 0.5%. Then the embryos can be used in the subsequent experiments. Two people will take part in one experiment, and one made the solution and treated the embryos, the other will take the picture and make the analysis.

### Chemical Treatment

The embryos at 4 hpf were examined under the stereomicroscope to assure they were at the same developmental stage. Then 30 embryos were randomly distributed into six-well plates (90 for each concentration) and exposed to E804 (0.5, 1, 2.5, 5, 10) dissolved in 3 ml of the fishing water for 96 hours. The control group was only treated with DMSO. The solutions were replaced and the dead embryos were removed at 24-hour interval. All tests were repeated three times.

As described above, embryos at 4 hpf exposed to different concentrations of E804 (0.5, 1, 2.5, 5, 10 µM) were recorded and photographed the phenotypic changes at 24, 48, 72, and 96 hpf. The hatching rate and the malformation rate at different time points was calculated and analyzed (The hatching rate = the hatching embryo/total number of the embryo * 100%. The malformation rate = the number of the malformation embryo/the number of the embryos alive * 100%). The body length of the zebrafish larvae at 96 hpf were measured and analyzed using ImageJ program. In subsequent experiments, the concentrations were selected according to the malformation rate. One concentration is below 10% malformation rate and one between 10 and 50%, one is about 50% and one is more than 50%.

### Assessment on the Cardiac Development Toxicity

*Tg(cmlc2:EGFP)* zebrafish line (Huang et al., 2003), which expressed EGFP driven by the cmlc2 promoter, was used to examined the effect of E804 on the morphology and function of the developing heart. After E804 exposure from 4 dpf, at least 10 tails of the zebrafish larvae at 72 hpf of each group were anesthetized, observed, and photographed using fluorescence microscope (Zeiss, Jena, Germany). The heart beating for 20 seconds was recorded, and the number was multiplied by 3 to calculate the heart rate. The area of pericardial edema and distance between the cardiac sinus venosus (SV) and bulbus arteriosus (BA) in the transgenic zebrafish were identified according to the GFP signal using ImageJ program. The experiments were conducted in three replicates and all tested were repeated for three times.

### Assessment of Vascular Changes

The *Tg(fli-EGFP)* zebrafish embryos (Hu et al., 2012), which expressed EGFP driven *fli* promotor were treated from 24 hpf with E804 until 48 hpf. After E804 treatment, at least 10 tails of zebrafish larvae of each concentration anesthetized by 0.16% tricaine were photographed using fluorescence microscope (Zeiss, Jena, Germany). Then number and total distance of the intersegmental vessels (ISVs) were counted and measured for assessment of the angiogenesis activity of E804 using ImageJ program. The experiments were repeated three times.

### Assessment of the Effect of E804 on the Developing Liver

The *Tg(l-fabp:EGFP)* zebrafish line expressing EGFP in the liver were used in the experiment (Her et al., 2003). After E804 treatment, the larvae (n = 10) anesthetized with 0.16% tricaine at 72 hpf were observed and photographed using a FSX100 Bio Imaging Navigator instrument (Olympus, Tokyo, Japan) focus on livers. The area and fluorescence intensity of the liver were assessed using ImageJ program as described (Zhang et al., 2016). The tests were repeated three times.

### Assessment of the Toxic Effect of E804 on Phagocytes

*Tg(Lyz : EGFP)* zebrafish line expressing EGFP in macrophages and neutrophils were used to assess the toxic effect by identifying the number of macrophage and neutrophil cells (Hall et al., 2007). After E804 treatment from 4 dpf until 72 hpf, microcopy was taken on larvae by using a fluorescence microscope (Zeiss, Jena, Germany) and the macrophage and neutrophil number of indicated area was counted using ImageJ program. The experiments were repeated for three times.

### Measurement of ROS Generation

Generation of ROS was examined at 72 hpf according to protocol of the ROS assay kit (Nanjing Jiancheng Bioengineering Institute, Nanjing, China). In brief, after E804 treatment, 10 larvae of the wild-type AB zebrafish line were treated with a 2-, 7-dichlorodihydrofluorescein diacetate (DCF-DA) solution (30 µg/ml) for 1 hour in dark at room temperature. After washed with PBS and anesthetized with 0.16% tricaine, microcopy was performed on larvae by using a fluorescence microscope (Zeiss, Jena, Germany). The fluorescence intensity of the larvae was identified by ImageJ program. The experiments were conducted in three replicates and all tests were repeated for three times.

### Apoptosis Assay

Apoptotic cells were detected by acridine orange (AO) staining in live larvae of the wild-type AB zebrafish line. Briefly, after E804 treatment (at 72 hpf), the larvae (n = 10) were washed with incubated with fish water for three times, incubated with 10 µg AO staining solution in the dark for 30 minutes at room temperature, and then washed with PBS for three times. Microscopy was performed on 10 tails of larvae anesthetized with 0.16% tricaine within 20 minutes by using a fluorescence microscope (Zeiss, Jena, Germany). The experiments were conducted in three replicates and all tests were repeated for three times.

### Assessment of Activity SOD and Level of MDA

After E804 treatment, 50 larvae of each concentration were collected together as a pool in cold saline. The larvae were homogenized and centrifugated to get the supernatants for the following assays. The coomassie blue protein binding method was used to determine the protein concentrations, and BSA was used as a standard control. The catalytic activity of total superoxide dismutase (SOD) and the levels of malondialdehyde (MDA) were assessed by using their respective commercial kit following the manufacture’s instruction (Nanjing Jiancheng Biotechnology Institute, China).

### Gene Expression Analysis

At 72 hpf, total RNA from 30 zebrafish larvae of each concentration were isolated using the TRIzol reagent (Invitrogen, Waltham, USA). The quality of the RNA was assessed based on the OD_260_/OD_280_ ratio. Then cDNA was synthesized using PrimeScript RT Kit in accordance to the manufacture’s instruction (TaKaRa Biotechnology, Dalian, China). Real-time PCR amplifications were performed using the SYBR Green mix (Takara, Dalian, China). Runs were carried out with three technical and three biological replicates and normalized using the housekeeping gene β-actin. 2^−△△Ct^ method was used to calculate the relative expression of the genes among groups. The primers used in this study are listed in **Table 1**.

**Table 1:** Primer pairs used in real-time quantitative PCR assay.

NO.	Gene	Forward primer (5′-3′)	Reverse primer (5′-3′)
1	L-FABP	acgtggcaggtttacgctcag	ttggaggtgatggtgaagtcg
2	scn5Lab	ttcaacatcggtctgctgct	tcgtgcattagtgccagtgt
3	BCL2	cactggatgactgactacctgaa	cctgcgagtcctcattctgtat
4	Bax	gacttgggagctgcacttct	tccgatctgctgcaaacact
5	Caspase 3	atgaccagggtcaaccataa	aagtacatctctttggtgagc
6	PPAR-α	ggcggacgattcggct	cgacagtattggcacttgttt
7	SOD	ggccaaccgatagtgttaga	ccagcgttgccagtttttag
8	TNF-α	gctggatcttcaaagtcgggtgta	tgtgagtctcagcacacttccatc
9	NRF-2	cacccaacatgaatcaactg	atttccgccatctgatgtaat

### Statistical Analysis

One-way ANOVA and Dunnett’s *t* test were used to analyze the results. The data were presented as mean ± SD. Statistical differences among groups were considered to be significant when *P* value was less than 0.05 or 0.01. The F values and the df (degrees of freedom) are listed in **Table 2**.

**Table 2:** The statistical data of F, degrees of freedom.

Fig.	Descriptions	F value	df （degrees of freedom）
**Figure 1**	Malformation rate	74.4	35
	Hatching rate	7.68	35
	Body length	219.9	179
**Figure 2**	Heart rate	37.63	49
	Pericardial area	24.62	49
	SV-BA distance	19.53	49
**Figure 3**	The number of the ISVs	91.86	49
	The Total length of the ISVs	53.5	49
**Figure 4**	Liver area	24.62	49
	Liver intensity	125	49
**Figure 5**	The number of macrophage and neutrophil	14.12	49
**Figure 6**	AO staining of the apoptotic cells	26.87	49
	Quantitative analysis of the apoptotic cells	47.21	49
**Figure 7**	SOD activities	14.55	14
	MDA content	27.18	19
**Figure 8**	Gene expression level	35.97	14

## Results

### Developmental Toxicity of E804 in Zebrafish Embryos

**Figure 1:**
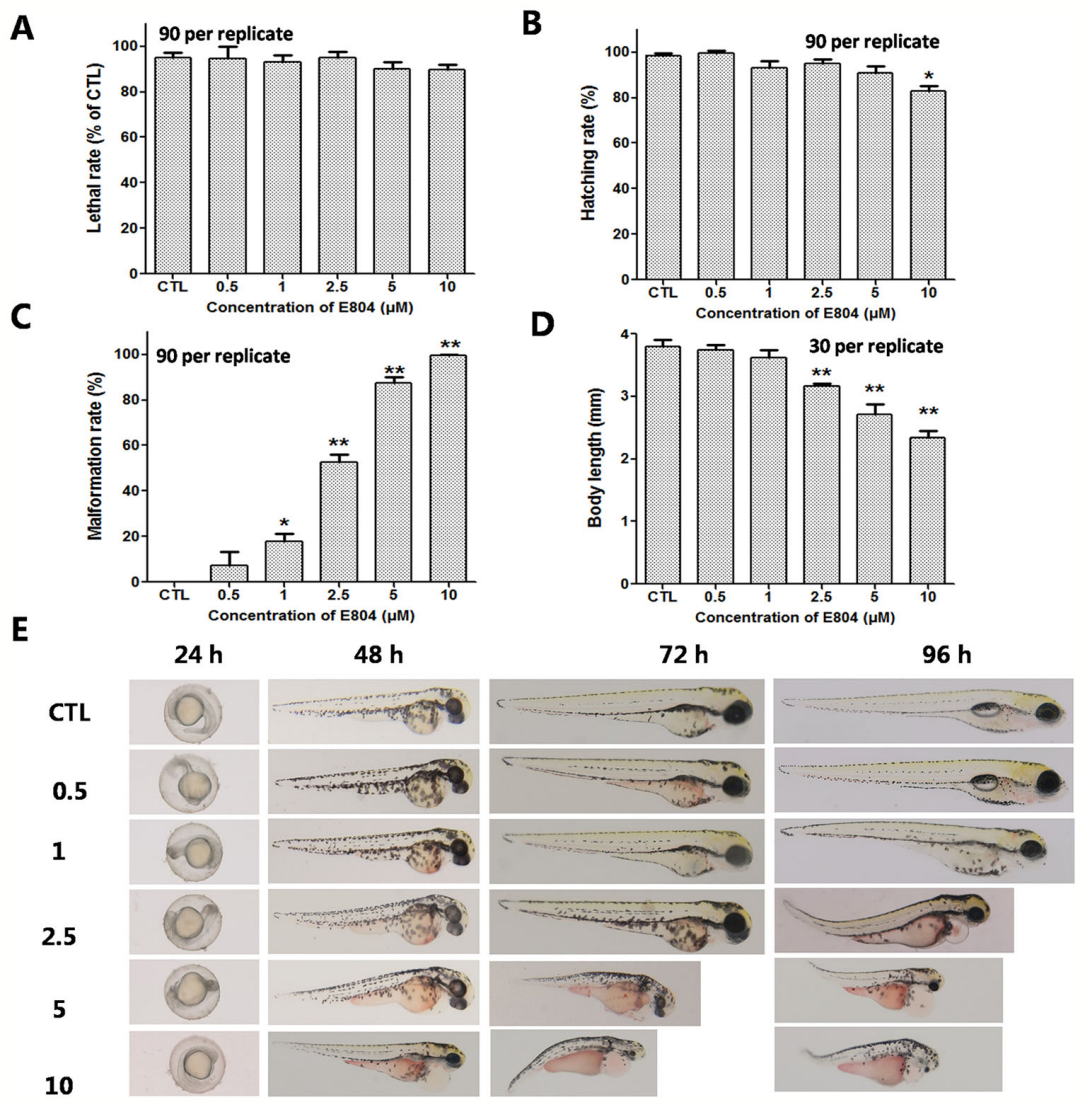
Developmental toxicity of E804 in zebrafish larvae (n = 3 replicates, N = 3 biological replicates, 90 embryos per replicate). **(A)** Lethal rate at 96 hpf (F = 52.3, df = 35) **(B)** Malformation rates at 96 hpf (F = 74.4, df = 35). **(C)** Hatching rates at 72 hpf (F = 7.68, df = 35). **(D)** Body length at 96 hpf (F = 56.72, df = 59). **(E)** The phenotypic changes of embryos at 24, 48, 72, and 96 hpf. The values are expressed as mean ± SD (*n* = 3). *Represents *p*-value less than 0.05 and **represents *p*-value less than 0.01. df means the degrees of freedom, F means F-distribution.

To investigate the developmental toxicity of E804 on zebrafish embryos, the mortality rates, malformation rate, and morphological abnormalities were recorded at 24, 48, 72, and 96 hpf (n = 3 replicates, 90 embryos per replicate). Our results indicated that there was no significantly decrease of the death rates following exposure with E804 under the concentration of 10 µM (*P* > 0.05) at 96 hpf (**Figure 1A**). The hatching rate was significantly decreased in the 10-µM E804-treated group (83%) compared with the control (*P* < 0.05) (**Figure 1B**), but there is no significant difference in other groups at 72 hpf (**Figure 1B**). Since solubility of E804 was poor (lower than 20 µM), we did not examined the effect of E804 on developmental toxicity of zebrafish larvae (Heshmati et al., 2013). However, severe malformation of all embryos at 96 hpf were observed in 1 (*P* < 0.05), 2.5 (*P* < 0.01), 5 (*P* < 0.01), and 10 µM (*P* < 0.01) E804 (**Figure 1C**) and the malformation rates present in a dose-dependent manner. The body length of embryos (n = 30) was significantly reduced in the 2.5 (*P* < 0.01), 5 (*P* < 0.01), and 10 (*P* < 0.01) µM E804-treated groups at 96 hpf (**Figure 1D**). Pericardial edema, curved body shape, absence of swim bladder and yolk retention were observed in the E804-treated groups (2.5, 5, and 10 µM) as shown in **Figure 1E**. At 96 hpf, there were pericardial edema observed of all the larvae at the concentrations of 5 and 10 µM E804.

### E804 Induces Cardiac Developmental Toxicity

**Figure 2:**
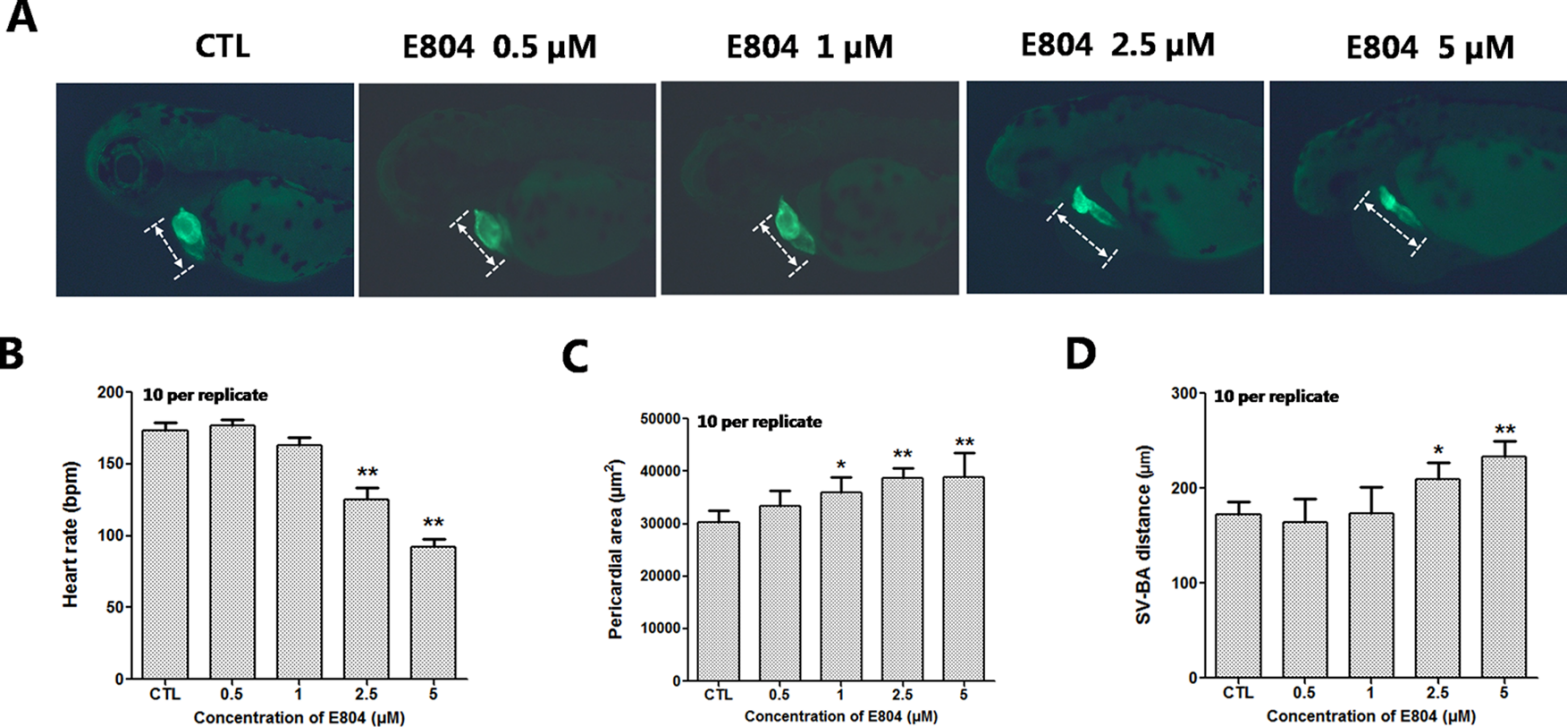
Effects of E804 on developing heart (n = 3 replicates, 10 per replicate). **(A)** Phenotypes of larvae of *Tg(cmlc2:EGFP)* lines. SV-BA distance is indicated by white row. **(B)** Heart rates at 72 hpf (F = 37.63, df = 29). **(C)** Pericardial area at 72 hpf (F = 24.62, df = 49). **(D)** SV-BA distance at 72 hpf (F = 19.53, df = 49). The values are expressed as mean ± SD (*n* = 3). *Represents *p*-value less than 0.05 and **represents *p*-value less than 0.01.

Heart rates and pericardial edema are used to detect the cardiac toxicity of zebrafish as previously reported (Bakkers, 2011; Jin et al., 2019). When assessing morphology of zebrafish embryo after E804 treatment, the most malformation abnormality was pericardial edema. To further investigate the cardiac developmental toxicity, we examined the heart phenotypes in the *Tg(cmlc2:EGFP)* transgenic line after E804 exposure (**Figure 2A**). The heart rates significantly reduced in 2.5 µM (125 ± 8 bpm) and 5 µM (92 ± 14 bpm) E804-treatment groups, significantly lower than those of the control (173 ± 12 bpm) (*P* < 0.01) (**Figure 2B**). The pericardial area in 1, 2.5, and 5 µM E804-treated groups significantly increased and presented in a dose-dependent manner (**Figure 2C**). In addition, the SV-BA distance E804-treated group increased significantly in the 2.5 (*P* < 0.05) and 5 µM (*P* < 0.01) (**Figure 2D**).

### Effects of E804 on Vascular Changes

**Figure 3:**
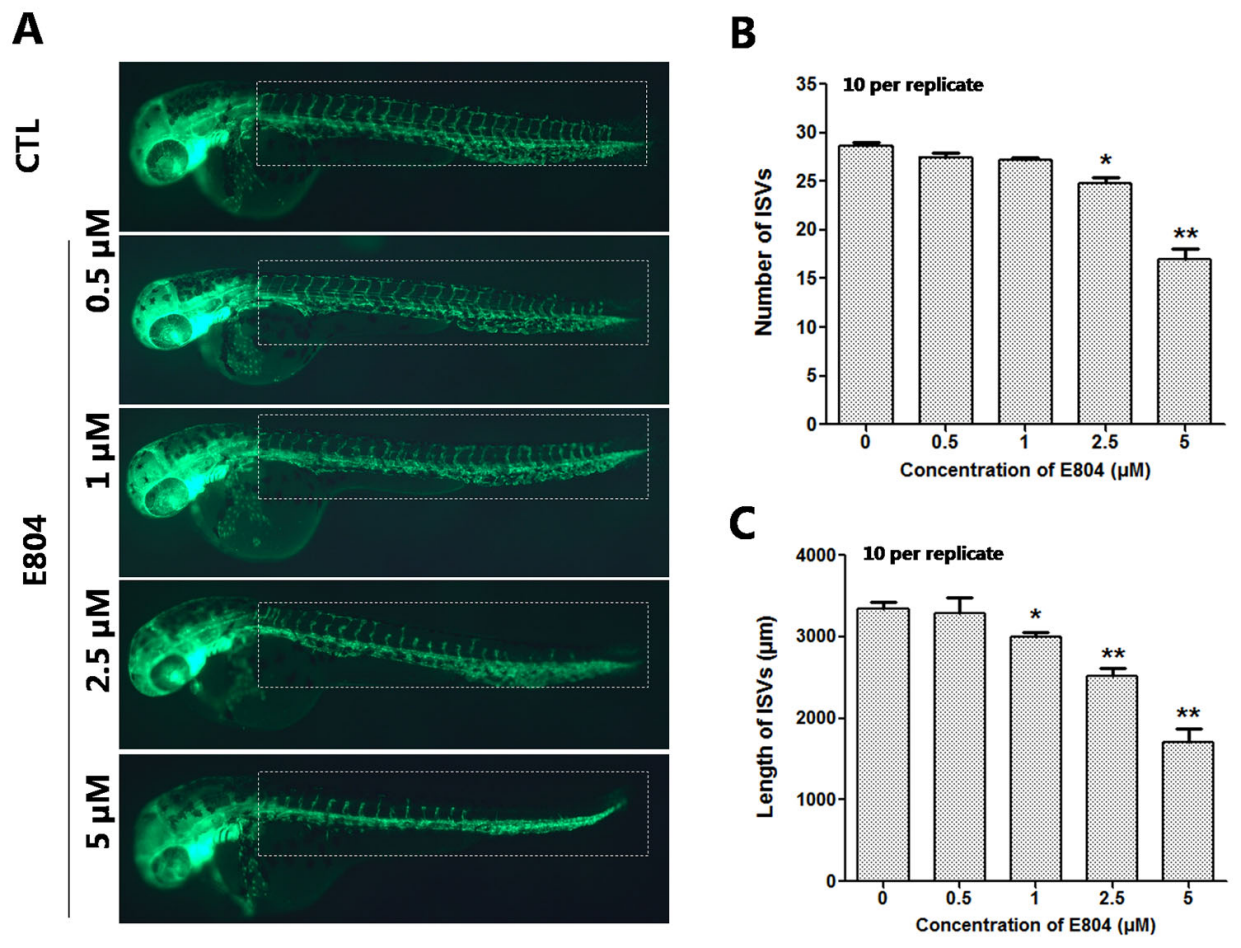
Effects of E804 on angiogenesis (n = 3 replicates, 10 embryos per replicate). **(A)** Phenotype of larvae of *Tg(fli-1:EGFP)* lines. The ISVs were counted and analyzed in white box regions. **(B)** The number of the ISVs at 48 hpf (F = 91.86, df = 49). **(C)** The total length of the ISVs at 48 hpf (F = 53.5, df = 49). The values are expressed as mean ± SD (*n* = 3). *Represents *p*-value less than 0.05 and **represents *p*-value less than 0.01.

Since numerous cardio- and cerebro-vascular diseases have angiogenesis deficiencies, we speculated that vascular blood maybe affected by E804. *Tg(fli:EGFP)* zebrafish embryos were used to assessed the toxic effects of vascular after E804 exposure (n = 3 replicates and N = 10 biological replicates). The number and the total length of the ISVs were recorded at 48 hpf after 24 hours treated with E804. ISVs developed normally and dorsally up from the dorsal aorta (DA) in the control, whereas significant loss or fragmentary of ISVs were observed in 2.5 (*P* < 0.05) and 5 µM (*P* < 0.01) E804-treated groups (**Figure 3A**). The number and the total length were significantly decreased in E804-treated groups and present a dose-dependents manner, which suggested that E804 could inhibit the vascular development (**Figures 3B, C**).

### Toxic Effects of E804 on the Development of the Zebrafish Liver

**Figure 4:**
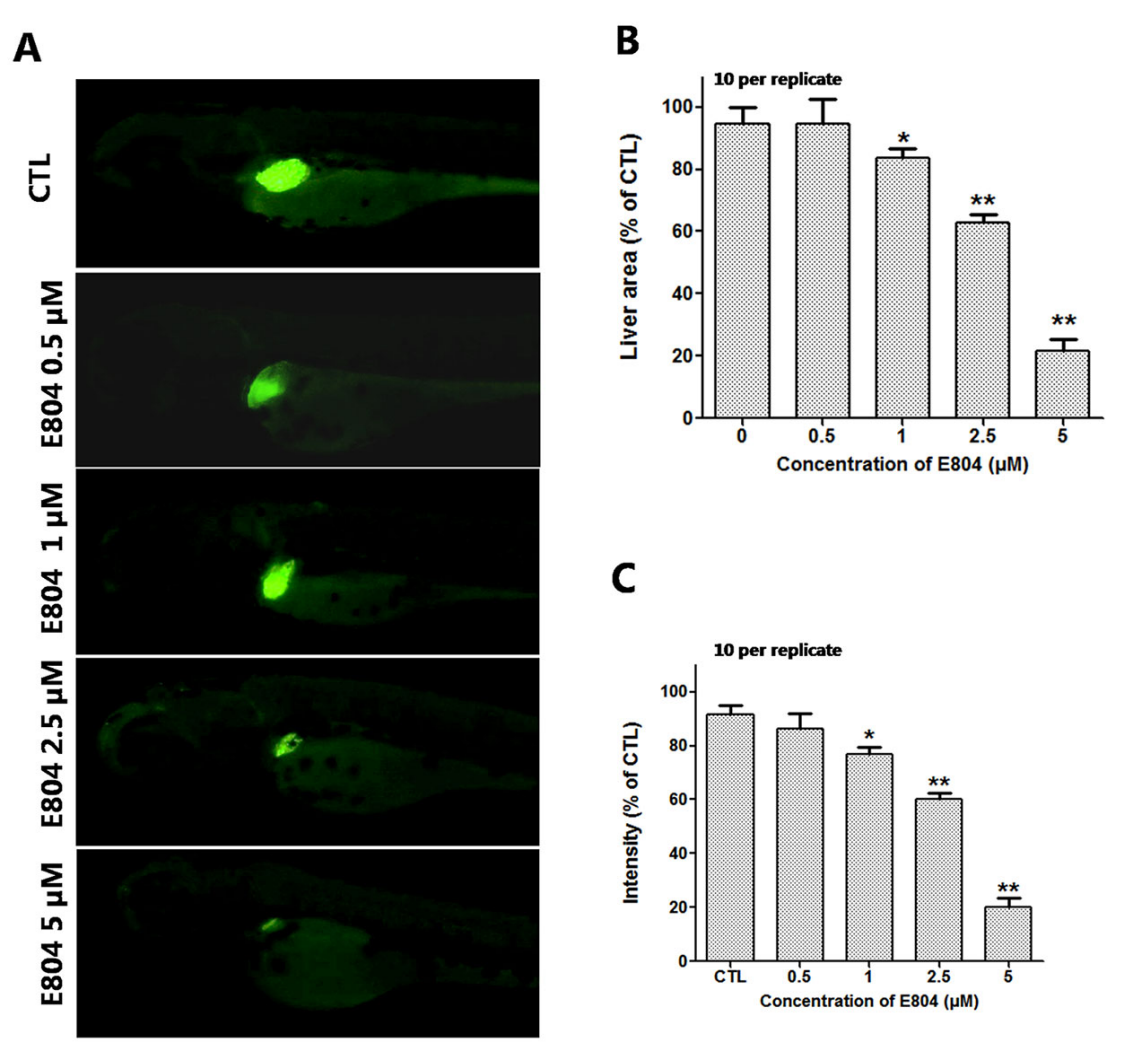
Effects of E804 on developing liver (n = 3 replicates, 10 embryos per replicate). **(A)** Phenotypes of larvae of *Tg(L-FABP : EGFP)* lines. **(B)** Liver area at 96 hpf (F = 24.62, df = 49). **(C)** Liver intensity at 96 hpf (F = 125, df = 54). The values are expressed as mean ± SD (*n* = 3). *Represents *p*-value less than 0.05 and **represents *p*-value less than 0.01.

To assess the toxicity of E804 on the development of zebrafish liver, *Tg(l-fabp-EGFP)* transgenic zebrafish embryos were used (n = 10 per group). As shown in **Figure 4**, the area and fluorescence intensity of the embryos’ liver both decreased and presented a concentration-dependent manner in E804-treated groups. The area of the embryo liver significantly decreased in the 1 µM (83.40 ± 2.20%), 2.5 µM (62.20 ± 2.02%), and 5 µM (21.50 ± 4.03%) groups (**Figure 4B**). And the fluorescence intensity of the liver in the 1, 2.5, and 5 µM E804-treatment groups decreased to 77.18 ± 2.38%, 61.18 ± 2.17%, and 19.33 ± 4.52% of that in the control group (*P* < 0.01) (**Figure 4C**).

### Effects of E804 on Number of Immune Cells

**Figure 5:**
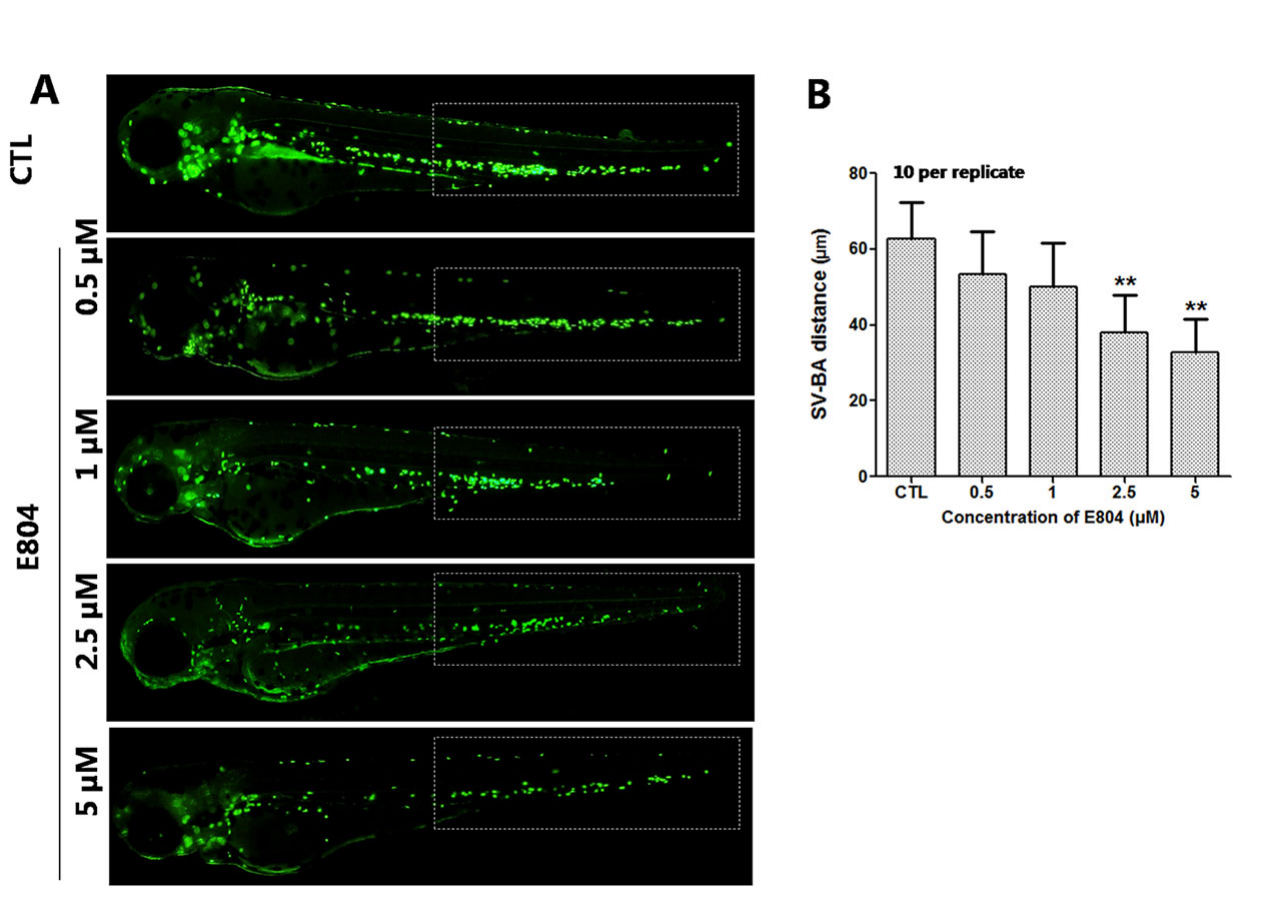
Effects of E804 on the macrophage and neutrophil (n = 3 replicates, 10 embryos per replicate). **(A)** Phenotypes of larvae of *Tg(Lyz : EGFP)* lines. The total number of macrophage and neutrophil was counted in white box regions. **(B)** Total number of macrophage and neutrophil in larvae at 72 hpf (F = 14.12, df = 49). The values are expressed as mean ± SD (*n* = 3). **Represents *p*-value less than 0.01.

The number of macrophages and neutrophils in indicated area in the *Tg(Lyz : GFP)* zebrafish larvae were counted to assess the toxic of E804 on immune system (n = 3 replicates and N = 10 biological replicates). It is shown that the number of the immune cells decreased significantly after E804 treatment at the 72 hpf. At the concentration of 2.5 and 5 µM E804-treated groups, the number of the macrophages and neutrophils reduced to 39.80 ± 2.51 and 32.10 ± 2.62, which was significantly lower than that of the control (62.32 ± 3.30) (*P* < 0.01) (**Figure 5**).

### E804 Induced ROS Generation

**Figure 6:**
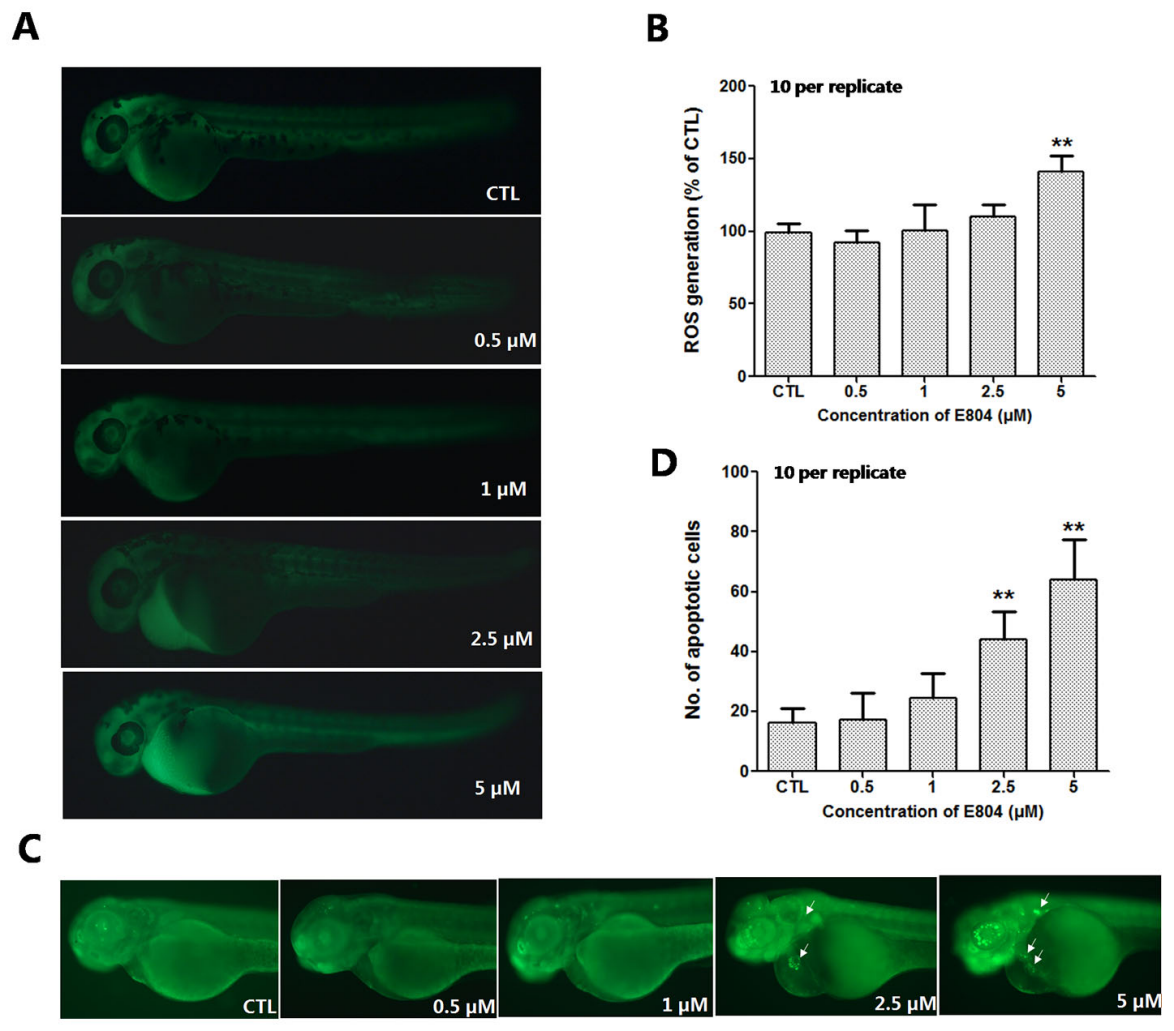
Effects of E804 on the ROS and apoptosis (n = 3 replicates, 10 embryos per replicate). **(A)** Fluorescence micrographs of ROS generation in zebrafish larvae at 72 hpf. **(B)** Quantitative analysis of ROS generation (F = 26.87, df = 49). **(C)** AO staining of the apoptotic cells. (White arrows indicate apoptotic cells in heart). **(D)** Quantitative analysis of the apoptotic cells (F = 47.21, df = 49). The values are expressed as mean ± SD (*n* = 3). **Represents *p*–value less than 0.01.

To further study the mechanism of developmental toxicity of E804 on zebrafish embryos, ROS generation was detected after E804 treatment at 72 hpf (n = 3 replicates and N = 10 biological replicates). As shown in **Figures 6A, B**, the embryos in the control group presented clear images, whereas the embryos in 5 µM E804-treated group generated stronger fluorescence signals (*P* < 0.01), which suggested that ROS generation took place after E804 exposure in zebrafish larvae.

### E804 Induced Apoptosis

AO staining was used to assess the effect of E804 treatment on apoptosis in zebrafish embryos (n = 3 replicates and N = 10 biological replicates). As shown in **Figure 6C**, the number of the apoptotic cells was significantly increased in 2.5 and 5 µM E804-treated groups (n = 10 per group), whereas there were no apoptotic cells observed in the control group. Apoptotic cells induced by E804 were mainly around the heart in E804-treated groups compared to the control group and the increase of the apoptotic cells number presented a dose dependent manner (*P* < 0.01) (**Figure 6D**).

### Effects of E804 on SOD Activity and MDA Levels

**Figure 7:**
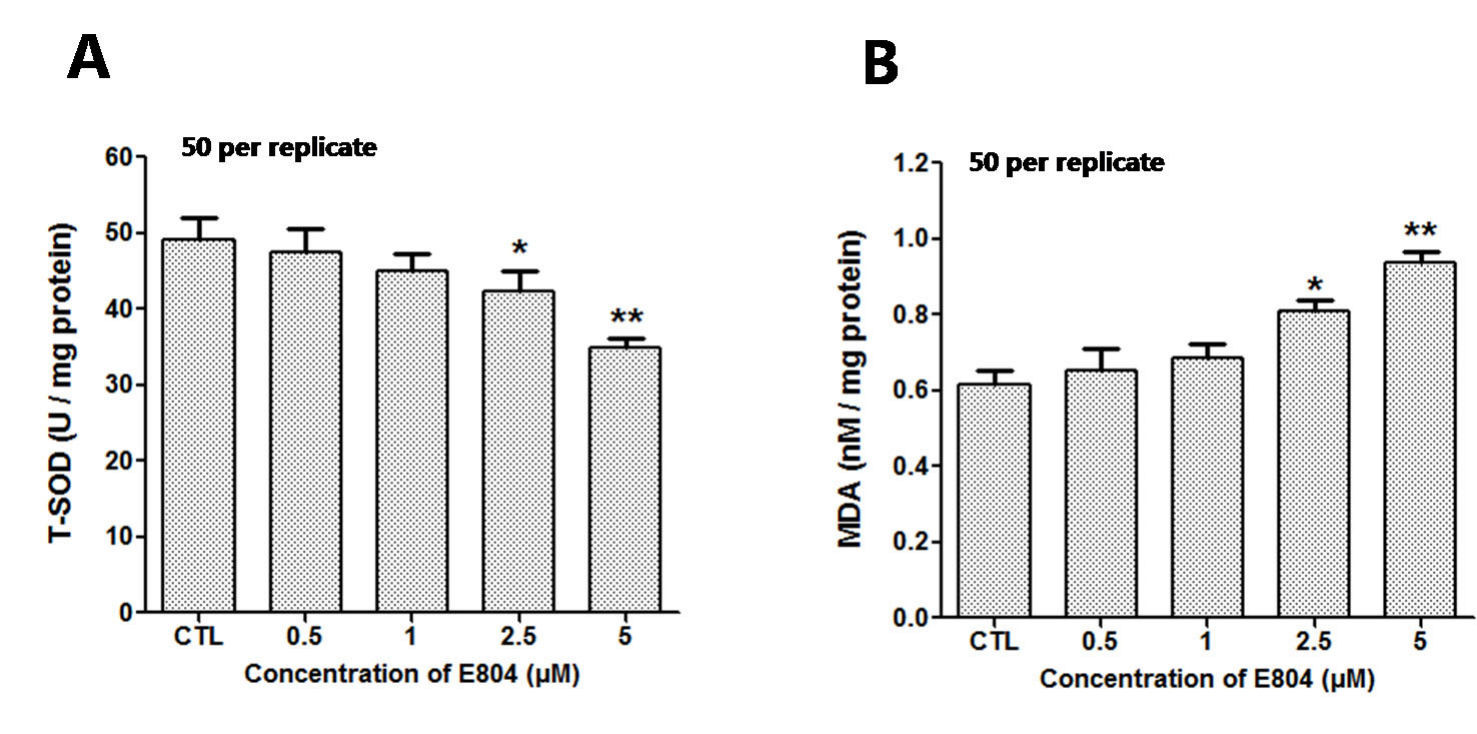
Effects of E804 on activities of SOD and the content of MDA in zebrafish at 72 hpf (n = 3 replicates, 50 embryos per replicate). **(A)** SOD activities (F = 14.55, df = 14). **(B)** MDA content (F = 27.18, df = 19). The values are expressed as mean ± SD. *Represents *p*-value less than 0.05 and **represents *p*-value less than 0.01.

After E804 treatment, the effects of E804 on SOD and MDA in larvae were detected (n = 3 replicates, 50 embryos per replicate). The results showed that the activities of SOD significantly decreased in 2.5 (*P* < 0.01) and 5 µM (*P* < 0.01) E804-treated groups and exhibited a concentration-dependent manner, whereas the MDA levels were significantly increased in 2.5 (*P* < 0.01) and 5 µM (*P* < 0.01) E804-treated groups (**Figure 7B**). The results showed that the antioxidant capacity decreased and the lipid peroxidation took place after E804 exposure.

### Analysis of Expression of Different Genes

**Figure 8:**
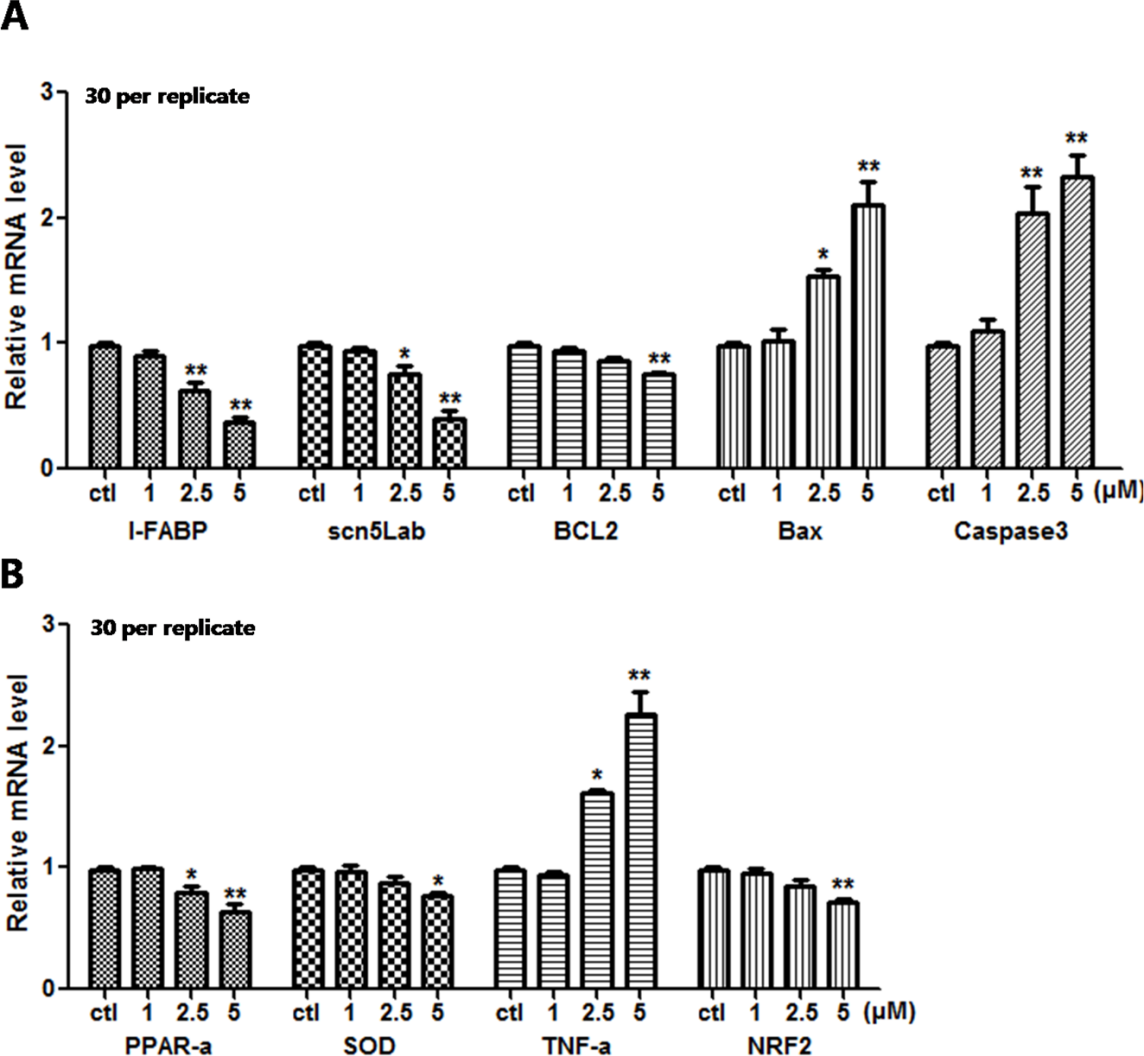
Effects of E804 on gene expression at 96 hpf (n = 3, 30 embryos per replicate). The mRNA expression level of L-FABP, scn5Lab, BCL2, Bax, Caspase 3 **(A)**, PPAR-α, SOD, TNF-α, and NRF-2 **(B)**. The values are expressed as mean ± SD (F = 35.97, df = 14). *Represents *p*-value less than 0.05 and **represents *p*-value less than 0.01.

Since apparent apoptosis and ROS generation were observed in the larvae after E804 treatment. To further study the molecular mechanism of toxicity induced by E804, the relative mRNA expression of different genes relative to oxidative stress, apoptosis were examined using real-time PCR (n = 3 replicates, 30 embryos per replicate) (**Figure 8**). qPCR data showed that the expression level of L-FABP was significantly decreased in 2.5 and 5 µM E804-treated groups at 72 hpf (*P* < 0.01). These results were consistent with the reductions of liver fluorescence in *L-FABP: EGFP* transgenic zebrafish embryos. Sodium channel-related gene (scn5Lab) expression level, which is required for zebrafish cardiogenesis, was significantly decreased in both 2.5 (*P* < 0.05) and 5 µM (*P* < 0.01) E804-treated groups. The decreased expression of Bcl-2 was observed in 5 µM (*P* < 0.01) E804-treated groups. The expression of Bax was markedly significantly increased in 2.5 (*P* < 0.05) and 5 µM (*P* < 0.01) E804-treated groups. The expression of Caspase 3 was notably increased in 2.5 and 5 µM (*P* < 0.01) E804-treated groups. PPAR-α expression was significantly decreased in both 2.5 (*P* < 0.05) and 5 µM (*P* < 0.01) E804-treated groups. The mRNA expression level of TNF-α was significantly increased in 2.5 (*P* < 0.05) and 5 µM (*P* < 0.01) E804-treated groups. The mRNA expression levels of NRF-2 and SOD was significantly decreased in 5 µM (*P* < 0.01) E804-treated groups. However, the expression levels of all genes were insignificantly changed in the 1 µM E804-treated groups.

## Discussion

Recent years, zebrafish have been widely accepted as an ideal alternative vertebrate animal model for *in vitro* assessment of toxicity of chemicals, organic pollutants, and nanoparticles.(Teraoka et al., 2003; Veldman and Lin, 2008). It is highly conserved of the development process in zebrafish compared with other vertebrates and is easy to observe this process (Lele and Krone, 1996; Tal et al., 2017). To assess the developmental toxicity of E804 on zebrafish embryo/larvae, we used wild type AB and several transgenic zebrafish lines in our study. The transgenic zebrafish lines are labeled with the GFP in liver, heart, blood vessels or immune cells. So it can be easy and direct to record the toxic effects of E804 on different organs or certain types of cells at different development stage of the embryos under the microscope. So far, there are more investigations about the anti-tumor or anti-angiogenesis activities, but little is known about its toxicities, especially the developmental toxicity. Here we aimed to study the toxic effects of E804 on development of the zebrafish embryos/larvae. Our results showed that E804 significantly affected the development of the zebrafish larvae especially the heart and liver. Further studies showed that generation of ROS and subsequent activation of apoptotic pathway might be the underlying mechanism of the developmental toxicity induced by E804.

Upon E804 treatment, pericardial edema, curved body shape, swim bladder deficiency, and yolk retention were observed at the concentration of 2.5 and 5 µM at 96 hpf. E804 treatment at 10 µM dose can decreased the hatching rate. Furthermore, the toxic effects of E804 on the developing heart, liver, blood vascular, and phagocytes in zebrafish embryos were examined. The heart rate was reduced and the pericardial area and SV-BA distance were increased in E804-treated groups, denoting cardiac dysfunction and structural abnormalities of the heart. The present study also showed E804 can lead to the blood vessel loss which is in line with previous studies (Chan et al., 2012; Shin and Kim, 2012), which may partly lead to the cardiac dysfunction. The mRNA expression level of scn5Lab, which is required for zebrafish cardiogenesis, was increased in E804-treated group and also indicated that the heart was damaged by E804. Previous reports also shows that pericardial edema, reduction of the heart rate, and smaller of the heart were observed in zebrafish which is deficient in scn5Lab gene (Bennett et al., 2013; Zhu et al., 2019). The decreased fluorescence area and intensity of the liver showed the liver damage in zebrafish larvae exposed to E804. The decrease of the macrophages and neutrophils number in indicated area of the zebrafish embryos showed the immune system was severely compromised. Given the solubility problem of E804, and according the abnormality rate, we selected of E804. However, the results also showed that E804 had severely developmental toxicity at lower concentration (< 2.5 µM).

Oxidative stress is considered to be an important reason for toxicity caused by compounds. The ROS level is tightly controlled and kept balance in the cells. Our study showed that the ROS level increased in zebrafish embryos after E804 treatment. Previous studies have shown that the induction of MDA indicated the oxidative damage of membrane lipids, while the SOD plays key defensive roles in antioxidant process (Yang et al., 2019). Our results showed that catalytic activity of MDA was highly increased, but the expression level of SOD was notably decreased by E804 in E804 treated groups. These results showed that E804 could induce oxidant damage and inhibit antioxidant progress in zebrafish embryos. To further study the apoptosis induced by high level of ROS after E804 treatment, the apoptotic cells were examined by acridine orange (AO) staining (Zhang et al., 2016). More apoptotic cells were observed in E804-treated groups, especially at the heart area, which were the most striking malformation in E804-treated groups.

To further study the underlying mechanism of developmental toxicity induced by E804, mRNA levels of genes correlated with oxidative stress and apoptosis were detected by real time RT-PCR. In our study, the mRNA levels of L-FABP, a member of the fatty acid binding protein family, and its target, peroxisome proliferator-activated receptor α (PPAR-α) were both down-regulated in a dose-dependent manner. L-FABP, highly expressed in hepatocytes and heart, is an important endogenous antioxidant during oxidative stress (Liu et al., 2008; Smathers et al., 2013). The deficiency of L-FABP could decrease the expression of PPAR-α by direct interaction of the proteins and provoke the oxidative stress (Velkov, 2013). Transcription factor NRF2 [nuclear factor (erythroid-derived 2)-like 2] is a defender against oxidative stress (Sun et al., 2019). The downregulation of NRF2 in E804 treated group suggested the antioxidant capacity of the embryos were induced. PPAR-α inhibition can activate the expression of TNF-α and then provoke more severe oxidative stress and induce apoptosis (Kim and Park, 2015). Furthermore, the mRNA expression level of Bcl-2, an anti-apoptotic gene, was inhibited by E804, whereas mRNA expression level of pro-apoptotic genes (Bax, caspase-3) were enhanced. It indicated the decrease of Bcl2/Bax ratio which demonstrated the occurrence of apoptosis in previous studies (Karmakar et al., 2016). Caspase3 can catalyze the cleavage of mane cellular proteins and then induce apoptosis (Untergasser et al., 2001). These results indicated that the oxidative stress and apoptosis played important roles in the developmental toxicity induced by E804 in zebrafish larvae.

In conclusion, E804 can induce developmental toxicity in zebrafish larvae. Our results showed that the E804 can induce pericardial edema, curved body shape, and short body length in zebrafish larvae. Moreover, E804 exerted toxic effects on the developing heart, liver, phagocytes, and blood vessel formation. Besides, our results indicated that E804 induced developmental toxicity by generation of ROS and apoptosis. These new findings will be helpful to understand the developmental toxicity induced by E804 and the underlying mechanisms. Further investigations in other animal models are needed to confirm our discoveries.

## Data Availability Statement

The datasets generated for this study are available on request to the corresponding authors.

## Ethics Statement

This study was carried out according to the guidelines and under the control of the Ethical Committee of the Biology Institute of the Shandong Academy of Sciences.

## Author Contributions

Conceived and designed the project: RW and KL. Performed the experiments: RW, XC and XW. Analyzed the data: RW and YZ. Wrote the manuscript: RW.

## Funding

Funding was provided by the National Science Foundation of Shandong Province (No. ZR2016YL009) and the National Key R&D Program of China (2018YFC1707300).

## Conflict of Interest

The authors declare that the research was conducted in the absence of any commercial or financial relationships that could be construed as a potential conflict of interest.
